# Assessing the Success of a Mineral Trioxide Aggregate and a Pre‐Mixed Bioceramic in Mature Teeth With Irreversible Pulpitis With Full Pulpotomy: A Randomized Clinical Trial

**DOI:** 10.1002/cre2.70090

**Published:** 2025-02-20

**Authors:** Sarang Suresh, Feroze A. Kalhoro, Priya Rani, Mahwish Memon

**Affiliations:** ^1^ Operative Dentistry & Endodontics, Faculty of Dentistry Liaquat University of Medical and Health Sciences Jamshoro Pakistan

**Keywords:** bioceramic, dental pulp disease, irreversible pulpitis, mineral trioxide aggregate, pulpotomy

## Abstract

**Objectives:**

The aim of this study is to compare the clinical and radiographic success of MTA versus EBRRM in pulpotomy of permanent teeth with irreversible pulpitis without apical periodontitis.

**Material and Methods:**

Clinical and radiographic assessments were conducted at baseline, 6 days, 6 weeks, and 6 months. After administration of anesthesia and coronal pulp removal, pulp was capped with MTA or Endo Sequence Bioceramic Root Repair, followed by restoration with a glass ionomer and resin composite.

**Results:**

The overall success rate for pulpotomy was 71.9%, with MTA and the bioceramic showing success rates of 32.8% and 39.1%, respectively. There was no significant relationship with the type of cavity and failure of pulpotomy.

**Conclusions:**

MTA and EBBRRM are both practical choices for pulpotomy and there is no notable difference between them in the success rate and pain level. EBBRRM may be more effective in Class 1 cavities than in Class 2 cavities.

## 1 Introduction

Irreversible pulpitis is a clinical diagnosis indicating the inability of inflamed pulp tissue to recover, typically caused by a localized inflammatory response to bacterial challenge, most commonly due to dental caries. Characteristic symptoms include exaggerated pain in response to thermal stimuli, which may persist after cessation of the provocation. However, the extent of inflammation cannot be accurately inferred from the size of the carious lesion, and severe spontaneous pain does not necessarily imply irreversible damage to the pulp, as the correlation between the clinical features of pulpitis and its histologic appearance remains controversial (Seltzer et al. 1963). Exposure of the pulp to oral microbiota during dental caries, trauma, or iatrogenic factors can lead to apical periodontitis, an inflammatory condition that affects the periapical region. According to Pak et al. (2012), apical periodontitis is present in 5% of the global population.

Pulpotomy, defined by the Glossary of Endodontic Terms of the American Association of Endodontists, involves the surgical removal of the coronal portion of the pulp to preserve the vitality of the remaining radicular pulp tissue. This approach is indicated when only a localized portion of the pulp is infected or inflamed. Although nonsurgical root canal treatment (NSRCT) has traditionally been the standard of care for irreversible pulpitis, advances in materials that preserve pulp viability have expanded our understanding of pulp biology (Zhang and Yelick 2010). Pulpotomy offers a conservative alternative, with reported success rates of 82%–95% in clinical trials (Simon et al. 2013; Solomon et al. 2015), compared to 87.5% in NSRCT (Galani et al. 2017). Despite this, NSRCT remains the preferred treatment among dentists, primarily due to its established protocol and reliability (Alqaderi et al. 2016). However, NSRCT is labor‐intensive, costly, often requiring multiple visits, and has a failure rate ranging from 24% to 66% (Asgary et al. 2013) due to factors such as inadequate obturation and post‐endodontic apical periodontitis, which occurs in 10% to 62% of cases (Persoon and Özok 2017). Preservation of pulp vitality offers significant advantages, including the maintenance of proprioceptive, defensive, and reparative functions, as well as vascularization and tooth sensitivity.

Mineral trioxide aggregate (MTA) has been a cornerstone material in endodontics, used for applications such as root perforation repair, apexification, root‐end filling, and pulp revascularization. MTA is composed of tricalcium silicate, tricalcium oxide, tricalcium aluminate, silicate oxide, and bismuth oxide, which contributes to its radiopacity. Reported success rates for MTA pulpotomy range from 84.6% to 90% (Alqaderi et al. 2014; Linsuwanont et al. 2017; Linu et al. 2017). Despite its predictability, MTA has notable shortcomings, including granular consistency that complicates handling, prolonged setting time, nonpredictable antimicrobial activity (Asgary and Kamrani 2008), discoloration due to the interaction of bismuth oxide with dentin collagen (Marciano et al. 2014), and potential leakage, surface disintegration, and loss of marginal adaptation (Parirokh and Torabinejad 2010).

To address these limitations, pre‐mixed bioceramic materials such as Endosequence Bioceramic Root Repair Material (EBRRM) have been developed. EBRRM, primarily composed of calcium silicate with tantalum pentoxide as the radiopacifier, is aluminum‐free and requires moisture from dentin for setting. These materials are available in putty and syringe forms, which simplify placement during procedures. Compared to MTA, use of bioceramics leads to faster setting times, superior handling, enhanced bioactivity, and reduced discoloration (Dong and Xu 2023). Their nanoparticle composition ensures better sealing, bioactivity, and adaptability to tooth structures, while promoting healing through hydroxyapatite formation and reducing postoperative inflammation (Raghavendra et al. 2017). Clinical studies report a 95% success rate for bioceramic putty in pulpotomy (Lei et al. 2019).

Although both MTA and bioceramic materials demonstrate high success rates in pulpotomy, comparative clinical studies evaluating their efficacy in treating irreversible pulpitis are limited. This study aims to address this gap by comparing the clinical efficacy of MTA and EBRRM pulpotomy in managing irreversible pulpitis in permanent teeth with fully developed roots.

The null hypothesis posits that there is no significant difference in clinical efficacy between pulpotomy procedures using MTA and EBRRM in fully developed permanent teeth with irreversible pulpitis but without apical periodontitis.

## 2 Methodology

This randomized clinical trial has been reported according to Preferred Reporting Items for Randomized Trials in Endodontics (PRIRATE) 2020 guidelines (Nagendrababu et al. 2020).

Modified clinical and radiological criteria were used to determine the success criteria. The following criteria were established for clinical success: no pain or discomfort after the initial 2 days of therapy, no pain upon palpation or percussion, a probing pocket depth of less than 4 mm, and no signs of inflammation or sinus tracts in the soft tissues surrounding the teeth. Radiographic success was defined as the absence of root resorption, furcal pathosis, or recent periapical pathosis as visible on the radiograph (Taha and Abdelkhader 2018).

The study used the randomized clinical trial (RCT) approach, and the trial registration number was NCT05853185. The research was carried out at the outpatient department of Operative Dentistry and Endodontics. Data were collected between October 12, 2021, and August 29, 2022. To select participants for the study, the researchers used a nonprobability sequential selection strategy.

Using the Kelsey approach on Openepi, the sample size for each population (MTA and EBRRM) was determined to be 32. The statistical power was set at 80%, with a 95% confidence level. This sample size was determined based on previous research on pulpotomy (Nosrat et al. 2013; Taha and Abdelkhader 2018).

Individuals older than 10 years of age who had irreversible pulpitis without apical periodontitis in maxillary and mandibular posterior teeth were included in this study. Teeth with signs of resorption, open apices, calcified or obstructed canals, perforations from dental procedures, root fractures, teeth deemed unrestorable, teeth unable to withstand extreme cold temperatures, teeth affected by sinus infections, or teeth with edema were all excluded.

On October 11, 2021, the study was approved, and ethical permission was granted vide letter (LUMHS/REC/‐93). Before the experiment began, all participants were provided with thorough explanations about the nature and goal of the study, after which their informed consent was obtained. To preserve data confidentiality, a procedure of anonymization was implemented, in which unique OPD numbers were allocated to everyone.

Participants were recruited, with a primary focus on people on the faculty of dentistry's waiting list. The researchers recorded demographic information, complaints, and clinical examination findings. The diagnosis of irreversible pulpitis was made when patients experienced lingering pain lasting at least 10 s following a cold test using a cold spray on a cotton bud, with the pain rated above 4 on a visual analogue scale.

Following administration of anesthesia, isolation, and caries‐driven access cavity, the decision was made to surgically remove pulp completely from the pulp chamber till root canal orifices. Hemostasis was obtained by immersing cotton in a 5% sodium hypochlorite for 10 min. If hemostasis could not be achieved within this time frame, the patient was excluded from the study and managed with NSRCT. The materials were used in accordance with the manufacturer's specifications, and the access cavities were provisionally restored with resin‐modified glass ionomer RMGIC.

Participants were randomized using a computer‐generated randomization sequence. The allocation concealment was maintained by using sealed, opaque envelopes, which were prepared by an independent researcher not involved in the study. Each envelope contained the assignment to either the MTA or EBRRM group, and it was opened only after the patient consented to participate in the study.

A pre‐established form was used to record patient data. Postoperative discomfort, edema, sinus, fistula presence, and tooth mobility were all evaluated 6 days, 6 weeks, and 6 months after the treatment. Periapical radiographs using sensor positioners were used for radiographic examination during these time intervals to evaluate the potential presence of radiolucency. In cases where postoperative failure occurred, such as persistent pain, swelling, or the development of periapical radiolucency, patients were managed with root canal therapy or extraction, depending on the clinical situation. These cases were documented separately and excluded from the final success rate calculations, ensuring transparency in the outcome analysis.

SPSS Version 20 was used to conduct data analysis. Gender, clinical and radiological success of the procedure, absence of pain, and sinus were computed in terms of frequencies and percentages. Continuous variables like the patient's age were assessed using statistical measures like the mean and standard deviation, or the median and interquartile range (IQR). The Shapiro–Wilk test was used to determine the data's normality. A chi square test was used to compare the clinical and radiological outcomes of the surgery between the two groups. To adjust for effect modifiers such as age, gender, and tooth number, stratification was used. This study used poststratification chi square and Fisher exact tests as statistical tests. The statistical significance was determined using a significance level of *p* = 0.05.

## 3 Results

This study included a total of 64 participants who met the inclusion criteria, with 100% follow‐up. Among them, 39 were males (60.9%) and 25 were females (39.1%), with ages ranging from 16 to 55 years and a mean age of 30.72 ± 10.868 years. The participants were also classified based on the American Society of Anesthesiologist (ASA), with 57 participants in ASA‐1 (89.1%) and seven participants in ASA‐2 (10.9%). The distribution of teeth used in the study is shown in Tables 1 and 2, and the type of cavity used was found to be Class 1 in 35 cases (54.7%) and Class 2 in 29 cases (45.3%). The results showed that overall, in 46 cases (71.9%), no failure occurred with pulpotomy, whereas in 16 cases (25%), clinical failure occurred, and in two cases (3.1%), clinical and radiological failure occurred. Pain was reported by 6.3% of participants on the 6th day, whereas 93.8% reported no pain. At 6 weeks and 6 months, 82.8% and 71.9% of participants reported no pain, respectively. There were no issues reported in terms of swelling, sinus, and mobility at any of the three time points (Table 1).

**Table 1:** PIRATE flow chart.

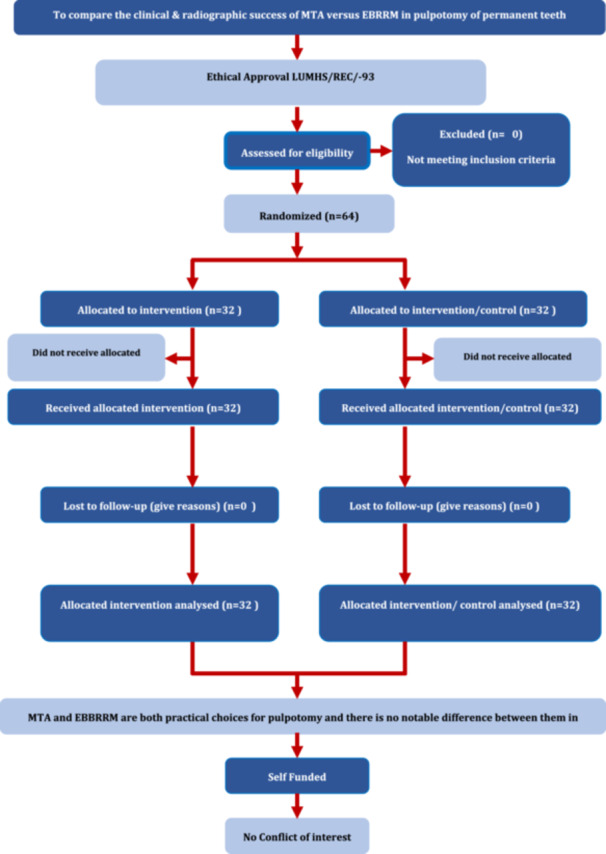

The normality of the data was tested using the Shapiro–Wilk test using a pulpotomy agent, and the results showed a *p*‐value of 0.001, indicating that the null hypothesis that the data follow a normal distribution can be rejected.

Based on the type of material, MTA showed a success rate of 32.8%, with no failure, and EBRRM showed a success rate of 39.1%, with no failure. The success rate for EBRRM was higher than that of MTA at all three time points, although the difference in success rates decreased with time. Additionally, the failure rate for both agents increased over time, with the highest number of failures observed at the 6‐month time point (Tables 2, 3, 4, 5, 6).

**Table 2:** Descriptive statistics.

Variables	Frequency	Percentage
Gender		
Male	39	60.9
Female	25	39.1
ASA class		
ASA–1	57	89.1
ASA–2	7	10.9
Tooth		
1st premolar	7	10.9
2nd premolar	9	14.1
1st molar	27	42.2
2nd molar	11	17.2
3rd molar	10	15.6
Cavity type		
Class 1	35	54.7
Class 2	29	45.3
Outcome on the 6th day		
Failure	4	6.3
Success	60	93.8
Outcome at the 6th week		
Failure	11	17.2
Success	53	82.8
Outcome at the 6th month		
Failure	18	28.1
Success	46	71.9
Type of failure		
No failure	46	71.9
Clinical failure	16	25
Clinical and radiological failure	2	3.1
Pain on the 6th day		
No	60	93.8
Yes	4	6.3
Pain at the 6th week		
No	53	82.8
Yes	11	17.2
Pain at the 6th month		
No	46	71.9
Yes	18	28.1
Periapical pathology at the 6th month		
No	62	96.9
Yes	2	3.1

**Table 3:** Chi square test for outcome on the 6th day in terms of the pulpotomy agent.

			Pulpotomy agent		Total	Chi square
			MTA	EBBRRM		0.302
Outcome on the 6th day	Failure	Count	3	1	4	
		% of total	4.7%	1.6%	6.2%	
	Success	Count	29	31	60	
		% of total	45.3%	48.4%	93.8%	
Total		Count	32	32	64	
		% of total	50.0%	50.0%	100.0%	

**Table 4:** Chi square test for outcome at 6th week in pulpotomy agent.

			Pulpotomy agent		Total	Chi square
			MTA	EBBRRM		0.740
Outcome at the 6th week	Failure	Count	6	5	11	
		% of total	9.4%	7.8%	17.2%	
	Success	Count	26	27	53	
		% of total	40.6%	42.2%	82.8%	
Total		Count	32	32	64	
		% of total	50.0%	50.0%	100.0%	

**Table 5:** Chi square test for outcome at the 6th month in terms of the pulpotomy agent.

			Pulpotomy agent		Total	Chi square
			MTA	EBBRRM		0.202
Outcome at the 6th month	Failure	Count	11	7	18	
		% of total	17.2%	10.9%	28.1%	
	Success	Count	21	25	46	
		% of total	32.8%	39.1%	71.9%	
Total		Count	32	32	64	
		% of total	50.0%	50.0%	100.0%	

**Table 6:** Chi square test for type of failure in MTA and EBRRM.

			Pulpotomy agent		Total	Chi square
			MTA	EBBRRM		0.273
Type of failure	No failure	Count	21	25	46	
		% of total	32.8%	39.1%	71.9%	
	Clinical failure	Count	9	7	16	
		% of total	14.1%	10.9%	25.0%	
	Clinical and radiological failure	Count	2	0	2	
		% of total	3.1%	0.0%	3.1%	
Total		Count	32	32	64	
		% of total	50.0%	50.0%	100.0%	

## 4 Discussion

Pulpotomy involves removing the coronal pulp while preserving healthy radicular pulp tissue. Though traditionally performed on primary teeth, it is also applicable to permanent teeth in specific cases (Ather et al. 2022; Akhlaghi and Khademi 2015; Linsuwanont et al. 2017; Shang et al. 2022). The significance of the procedure lies in preventing infection and inflammation spread while maintaining tooth function and structure. Pulpotomy offers benefits such as pain relief by reducing interstitial pressure (Nyerere et al. 2006), preservation of tooth structure by removing only the infected pulp, and cost‐effectiveness compared to root canal therapy or extraction (Simon et al. 2013).

This study evaluated the impact of cavity type on pulpotomy success, observing a trend linking cavity design to failures when EBRRM was used. EBRRM, a bioactive material composed of calcium silicate, zirconium oxide, and hydroxyapatite, promotes mineralized layer formation upon moisture exposure. However, moisture control and dentin preparation are critical. Inadequate preparation or excessive moisture can compromise EBRRM's setting, causing microleakage, bacterial ingress, or material fractures (Huang 2011; Wang et al. 2023). Class II cavities may present challenges in achieving a complete pulp chamber seal due to EBRRM's putty consistency, increasing failure risk (Igna 2021).

Consistent with prior research, MTA demonstrated effectiveness in pulpotomy. Studies report success rates exceeding 80% for MTA (Kiranmayi et al. 2022; Chandrasekhar et al. 2022), aligning with our findings. A comparative study by Qian et al. (2022) reported a 100% success rate for MTA and 94.4% for bioceramic materials. Adequate sealing with bioactive material and coronal restoration, alongside regular follow‐ups, remains essential for success (Sadaf 2020).

Our findings show pulpotomy as a viable treatment option for Class I and II cavities, with no significant differences in the success rates between ASA‐1 and ASA‐2 patients. Although MTA and EBRRM demonstrated comparable success rates regardless of cavity type, caution is advised when using EBRRM in Class II cavities due to the observed correlation between cavity type and failure.

From a theoretical perspective, this study provides evidence on pulpotomy success rates across cavity classes and agents, informing future models on influencing factors. Practically, the findings support the use of MTA or EBRRM for pulpotomy with similar success rates, irrespective of cavity type.

Future research should address this study's limitations, including its small sample size (64 participants) and short‐term follow‐up. Larger, long‐term studies are needed to validate these findings. Additionally, factors such as operator experience and patient oral hygiene warrant investigation. Future studies could explore the efficacy of other pulpotomy agents or compare a broader range of materials. Moreover, examination of the influence of variables like age, gender, and medical history on pulpotomy outcomes would enhance understanding.

## 5 Conclusion

According to the results, it can be concluded that both MTA and EBBRRM are practical choices for pulpotomy and there is no notable difference between them in the success rate and pain level. The findings suggest that both materials are effective in maintaining pulp vitality and preventing failure of the procedure, with no significant difference in the success rate between the two agents. The study also indicates that pulpotomy using MTA and EBBRRM is a safe and reliable procedure, with a low incidence of pain, swelling, sinus, and mobility reported at all three time points.

## Author Contributions

Sarang Suresh conceptualized the study, and contributed to the methodology, data collection, and formal analysis. Feroze A. Kalhoro supervised the research project, provided critical revisions, and contributed to the overall design and validation of the study. Priya Rani assisted with data collection and analysis. Mahwish Memon provided critical insights into the methodology and data validation.

## Conflicts of Interest

The authors declare no conflicts of interest.

## Data Availability

The data that support the findings of this study are available from the corresponding author upon reasonable request.
